# Sexual Behavior among Persons Living with HIV in Uganda: Implications for Policy and Practice

**DOI:** 10.1371/journal.pone.0085646

**Published:** 2014-01-23

**Authors:** Geofrey Musinguzi, Denis Bwayo, Noah Kiwanuka, Sheila Coutinho, Aggrey Mukose, Joseph Kabanda, Lilian Sekabembe, Fred Nuwaha

**Affiliations:** 1 Department of Disease Control and Environmental Health, School of Public Health, College of Health Sciences, Makerere University, Kampala, Uganda; 2 Civil Society Fund, Kampala, Uganda; 3 Department of Epidemiology and Biostatistics, School of Public Health, College of Health Sciences, Makerere University, Kampala, Uganda; Alberta Provincial Laboratory for Public Health/ University of Alberta, Canada

## Abstract

**Introduction:**

HIV epidemics are sustained and propagated by new cases of infection which result from transmission from infected persons to uninfected susceptible individuals. People living with HIV (PLHIV) play a critical role in prevention if they adopt safer sexual behaviors. This study estimated the prevalence of and factors associated with safer sexual behaviors among PLHIV seeking care from civil society organizations (CSOs).

**Methods:**

In a cross sectional study PLHIV were interviewed about their sexual practices, use of alcohol, HIV status of their regular sexual partners, desire for more children and about their socio-demographic characteristics. We calculated the proportion of PLHIV who abstained and consistently used condoms in the previous twelve months. Independent associations between safer sex and other variables were estimated using adjusted prevalence ratios (aPR) and their 95% confidence intervals (CI).

**Results:**

Of the 939 PLHIV, 54% (508) were either abstaining or using condoms consistently and 291 (31%) desired more children. The prevalence of consistent condom use among the sexually active was 41.3% (300/731). Consistent condom use was higher among PLHIV who: didn't use alcohol (aPR 1.30, CI 1.03–1.63); were educated about re-infection with a new strain of HIV (aPR 1.84, CI 1.08–3.12) and had regular sexual partner who was HIV negative (aPR 1.29, CI 1.05–1.57). Prevalence of abstinence was 22.2% (208/939). Abstinence increased with age from 9.4% among PLHIV <25 years to 40.5% among those >50 years. Abstinence was extremely low (2.5%) among PLHIV who were married.

**Conclusions:**

Effective interventions that reduce alcohol consumption among PLHIV are needed to avert HIV transmission, prevent acquisition of new HIV strains and STIs. In addition, strategies are needed to address needs of PLHIV who desire more children.

## Background

HIV/AIDS remains a global public health challenge. In 2011, The World Health Organization (WHO) estimated that 34 million people were infected with HIV of whom 2.5 million were newly infected and 1.7 million deaths were registered in the same year [1]. Sub-Saharan Africa is the region most affected, with nearly 1 in every 20 adults living with HIV [1].

In Uganda, HIV started to decline in the early 1990s and Uganda was applauded as a regional hero in the fight against HIV [2], [3]. The success story of sustained decline in HIV prevalence was explained by a combination of factors among which included strong political will and openness, and an intersectoral approach to HIV/AIDS prevention which vividly promoted the Abstinence, Be faithful and Condom use strategy (The ABC strategy) [4].

More recently, there is evidence that HIV prevalence and incidence are no longer decreasing [5], [6]. Uganda is listed among those countries that currently have stable or increasing infection rates, and this picture is also evident in the recent Uganda AIDS Indicator Survey (UAIS) 2011 [5]. According to UNAIDS (2011), 1,400,000 people are living with HIV/AIDS in Uganda [7]. Moreover, a review of the magnitude, trends and drivers of HIV/AIDS epidemic reveal a resurgence of risky sexual behaviors among Ugandan adults aged 15–49 years. An increasing proportion of Ugandans had sex with high risk partners, acquired multiple sexual partners, and inconsistently used condoms. This could partly explain the current (2011) HIV adult prevalence of 7.2% among the 15–49 year age group [5] up from 6.4% in 2005 [3].

HIV epidemics are sustained and propagated by new cases of infection which result from transmission from infected persons to uninfected susceptible individuals [8]. Given the natural history of HIV, many of the infected persons are not aware of their status until they have tested for HIV but even after testing, they play a critical role in prevention if they adopt safer sexual behaviors [9]. As a result, HIV/AIDS prevention efforts are increasingly targeting persons living with HIV/AIDS with prevention with positive programs (PwP) often called positive health, dignity and prevention (PHDP) that provide comprehensive HIV services [10].

With increased access to treatment for PLHIV, there has been a consequent decrease in mortality among PLHIV in care, improved wellbeing and subsequent increase in normal functioning including sexual activity [5], [11]. Therefore PLHIV in care continue to be at risk of acquiring new strains of HIV and transmitting HIV to others. Indeed, high-risk sexual behaviors continue to be reported in Uganda [12], [13].

Risky sexual behaviors have several facets. Tumukunde et al [12] found that ART experienced (PLHIV on ART) patients were significantly less likely than the naïve (PLHIV not on ART) to engage in risky sexual behavior in Uganda. In India, a study on prevalence and contexts of inconsistent condom use among heterosexual men and women living with HIV showed that one third of men and one fourth of women reported inconsistent condom use with regular sexual partners. The reported prevalence of inconsistent condom use was attributed to: the beliefs that condoms were unnecessary in HIV-positive sero-concordant relationships; lack of sexual satisfaction with condoms; the desire to have children; husbands using alcohol; depression and anxiety; fear that disclosure of HIV status would bring marital discord and family shame and inadequate counseling by health care providers [14]. Sarna et al [15] reported that risky sexual behaviors decreased with ART and a substantial proportion of PLHIV on ART continued to have unsafe sex, even with partners known to be HIV negative [16]. A Côte d'Ivoire study reported a short-term increase in unsafe sexual behaviors after ART initiation [17]. In India, safer sexual practices of consistent condom use were attributed to the feeling of personal responsibility to protect the health of the partner, desire to prevent acquisition and/or transmission of sexually transmitted infections, and the belief that condoms were needed for antiretroviral therapy to be effective [14].

Understanding sexual behaviors among PLHIV can provide useful insights to guide efforts in preventing further HIV transmission, and helps to enable PLHIV to lead healthy and responsible lives [18].

## Methods

### Study setting

The study was conducted among adult (18 years and above) persons living with HIV/AIDS and receiving care from civil society organizations supported by the Civil Society Fund (CSF). The CSF was established under the Uganda AIDS Commission to support the objectives of the National Strategic Plan for HIV/AIDS and Orphans and Other Vulnerable Children (OVC). The goal of the CSF is to ensure that civil society provision of prevention, care, treatment, and support services in HIV/AIDS and OVC are harmonized, streamlined, effective, and in support of the Government of Uganda (GOU) National Strategic Plan, National Priority Action Plan, and other national plans and policies. The map (Figure S1) shows the zonal distribution of CSF sub grantees in the various regions of Uganda that provide care to people living with HIV/AIDS. These include The AIDS Support organization (TASO), the Joint Clinical Research Center (JCRC), the Infectious Diseases Institute (IDI) and the AIDS Information Center (AIC). TASO operates in 10 districts, JCRC in 16 districts, AIC in 8 districts and IDI in 6 districts. From each of the 8 sub regions illustrated in the map (Figure S1), we selected one CSO to account for geographical inclusiveness and ethnic diversity. Administratively, Uganda is divided into 112 districts with an estimated population of 34 million people most of whom depend on agriculture for income and livelihood [19]. From each of the eight sub-regions one district hosting a CSO was selected. As depicted in the map (Figure S1), CSOs are geographically present in fewer districts. Actually most of the CSOs serve more than one district and in some cases a whole sub-region. The CSO hosting districts included in this study were; Masindi, Mubende, Masaka, Mbarara, Tororo, Soroti, Lira, and Arua. The corresponding CSOs selected were TASO Masindi, IDI Mubende, TASO Masaka, AIC Mbarara, JCRC Tororo, JCRC Soroti, AIC Arua and IDI Lira. All the selected districts are largely rural with a small proportion of the population living in urban centers.

### Study design

This was a cross-sectional study utilizing quantitative survey methods of data collection. The questionnaire used for data collection was adopted with modifications [12], [14], [16], pretested, translated and back translated into the 8 major languages for each of the studied districts. The main domains of the study tool included socio-demographic characteristics, sexual behaviors of PLHIV, disclosure and HIV status of sexual partners, alcohol and substance use, health service factors, child factors and ART status. The socio demographic details included: place of residence, age, sex, marital status, education, and employment status. Sexual behaviors enlisted included whether PLHIV had sexual intercourse in the past 12 months, number of sexual partners (regular and non regular), and condom use. Disclosure and HIV status questions asked were; whether regular sexual partners were aware of HIV status of PLHIV, how they got to know, whether PLHIV knew the HIV status of their regular sexual partners, the HIV status of the regular sexual partner and whether assistance was needed to disclose. The child factor component investigated whether PLHIV had ever given birth, whether they had biological children, how many were alive, whether they desired any/more children, whether they had been pregnant or caused a pregnancy in the past 12 months and whether these pregnancies were intended or not. The questions on ART were about whether they were receiving ART or not, how long they had been on ART or Opportunistic Infection (OI) prophylaxis and self rating of health status since starting ART.

### Sampling of study subjects

This study was conducted over a 10 day period in the months of August and September 2012. PLHIV's medical files scheduled for each of the 10 clinic days constituted the sampling frame from which 12 study subjects per facility per day were selected by simple random sampling. In Uganda, PLHIV in care constitute 70% women and 30% males, 54% of whom are ART experienced [7], [20]. On average, an active HIV clinic at a CSO based in rural district attends to 100 clients a day. To ensure adequate representation of sub-populations for stratified analysis, scheduled client files were sorted by gender and ART status. Consequently, numbers (1 or 2) were assigned to each file in each stratum and corresponding numbers were written on pieces of papers which were folded, churned and picked randomly from the bowl by eligible study subjects without replacement. All eligible study subjects with a 1 were enrolled for the study. Figure S2 illustrates the criteria used to sample study subjects at a CSO facility.

### Sample size and data collection

We used the Kish formula [21] to determine the sample size with α of 0.05, p = 0.5, and d = 0.05 (5%) and estimated a sample size of 384. We factored in a non response rate of 15% and a design effect of 2 [22] to compensate for clustering and to generate the minimum required sample of 884 study participants. The data was collected by trained research assistants who were fluent in both English and the local languages spoken in the selected districts.

### Ethics statement

Ethical approval was granted from the Makerere University School of Public Health Higher Degrees-Research and Ethics Committee and the Uganda National Council for Science and Technology. All selected CSOs were asked for permission to have their organizations participate. The Helsinki principles of voluntary participation were followed and all study participants provided written informed consent.

### Definition of variables

#### Safer sexual behavior

People Living with HIV were classified as practicing safer sexual behavior if they consistently used a condom during every sexual act or abstained from sex in the 12 months preceding the study.

#### Consistent condom use

Was defined as a practice of always using condoms during every sex act in the twelve months preceding the study.

#### Abstinence

PLHIV were classified as abstaining if they reported that they did not have any sexual intercourse within the 12 months preceding the study

#### Regular sexual partner

A spouse or cohabiting sexual partner.

### Data analysis

Completed data tools were edited daily, compiled by the field team supervisor, and logged through counting and locating them into transportation boxes. Data capture screens with in-built checks for consistency, logical flow, range and accuracy of data were designed in EPIDATA version 3.1 and were used for electronic data capture. Electronic data was transferred from EPIDATA to Stata® version 12 (StataCorp, College Station, TX) software for statistical analyses. In order to adjust for clustering, robust standard errors and cluster option in STATA were utilized. Because abstinence and condom use are distinct i.e. one cannot abstain and use condoms, two separate models were employed to assess their prevalence and related factors. However, to estimate the overall prevalence of safer sexual behaviors, a composite (consistent condom use or abstinence) was constructed. Comparisons of variables across each other and by sub-categories of the outcome variable were conducted by chi-square and Fisher's exact test for categorical variables. Prevalence ratios (PRs) were used as a measure of association to avoid overestimation of the risk – because the outcome prevalence was >10%. Adjusted PRs were calculated through log-binomial regression models. Both unadjusted and adjusted PRs were estimated with associated 95% confidence intervals. Multivariable models were adjusted for potential confounders which were added to models based on biological plausibility as well as predetermined statistical significance of 0.15 at bivariate analysis. For all tests, a p-value of <0.05 was taken as statistically significant.

## Results

### Characteristics of the study participants

Nine hundred sixty (960) participants consented to participate in the study. Of these 418 (43.5%) were males and 542 (56.5%) were females. Twenty one (2.2%) were excluded from analysis because of missing data. Of the 939 responses analyzed, 44% were males and 56% were females. Table 1 describes selected characteristics of the study participants, stratified by gender. About 80% of the study subjects were aged 30 years and above. The mean age was higher among males (40.8%) than females (36.4%). Nearly a third (29.4%) completed secondary school education and above, majority (62.4%) resided in rural areas, with 44.2% having some form of employment or occupation. The median monthly average income was less than US$24 and males earned more money than females [median (IQR) average monthly income was US$34.8(US$11.6–US$77.4) versus US$19.3(US$7.7–US$38.7) respectively].

**Table 1:** Selected Characteristics of the study population (n = 939).

Characteristics	N	Male	Female	P value*
**Total**	939	418 (43.5%)	542 (56.5%)	
**Age** (years)				
Mean (SD)	38.1 (9.9)	40.8 (9.7)	36.2 (9.5)	<0.0001
18–24	64 (6.8%)	10 (2.4%)	54 (10.3%)	
25–29	20(12.8%)	33 (8.0%)	87 (16.5%)	
30–39	354 (37.7%)	154(37. 2%)	201 (38.2%)	
40–49	280(29.8%)	144 (34.9%)	136 (25.9%)	
50+	121(12.9%)	73 (17.5%)	48 (9.1%)	<0.0001
**Highest Education level**				
None	93(9.9%)	20 (4.8%)	73 (13.9%)	
Primary	570 (60.7%)	269 (65.1%)	301 (57.2%)	
Secondary	213 (22.7%)	87 (21.1%)	126 (24.0%)	
University/Tertiary	63 (6.7%)	37 (9.0%)	26 (4.9%)	<0.0001
**Residence**				
Urban	353(37.6%)	141 (34.1%)	212 (40.3%)	
Rural	586 (62.4%)	272 (65.9%)	314 (59.7%)	0.032
**Employment**				
Yes	412 (44.2%)	213 (52.1%)	199 (38.0%)	
No	520 (55.8%)	196 (47.9%)	334 (62.0%)	<0.0001
**Marital status**				
Married/living together	555(59.2%)	323 (78.2%)	232 (44.2%)	
Divorced/separated	167 (17.8%)	49 (11.9%)	118 (22.5%)	
Widowed/widower	165 (17.6%)	23 (5.6%)	152 (27.1%)	
Never married/lived together	51 (5.4%)	18 (4.3%)	33 (6.2%)	<0.0001
**Duration since knowing one**'**s HIV results**				
<1year	143 (15.2%)	74 (17.9%)	69 (13.1%)	
1–2 years	201 (21.4%)	96 (23.2%)	105 (18.0%)	
2.1–5 years	245 (26.1%)	110 (26.6%)	135 (25.7%)	
>5 years	350 (37.3%)	133 (32.3%)	217 (43.2%)	0.02
**On ARVs**				
Yes	552 (59.0%)	258 (62.9%)	294 (55.9%)	
No	384 (41.0%)	152 (37.1%)	232 (44.1%)	0.03
**Number of children PLHIV have ever had**				
0	49(5.2%)	21(5.1%)	28(5.3%)	
1	78 (8.3%)	25(6.1%)	53(10.1%)	
2	110(11.7%)	40(9.6%)	70(13.3%)	
3	118(12.2%)	46(11.1%)	72(13.7%)	
4	152(16.2%)	68(16.5%)	84(16.0%)	
5 or more	432(46.4%)	213(51.6%)	219(41.6%)	0.021
**Desire for more children**				
Yes	291 (31.3%)	143 (34.7%)	148 (28.5%)	
No	640 (68.7%)	269 (65.3%)	371 (71.5%)	0.043
**Number of desired children**				
One	109(39.1%)	43(31.9%)	66(45.8%)	
Two	115(41.2%)	64(47.4%)	51(35.4%)	
3or more	55(19.7%)	28(20.7%)	27(18.8%)	0.048
**Number of sexual partners in past 12 months**				
0	208 (22.2%)	47 (11.4%)	161 (30.6%)	
1	585 (62.2%)	259 (62.7%)	326 (62.0%)	
2+	146 (15.6%)	107 (25.9%)	39 (7.4%)	<0.0001
**Self rating of current health status**				
Better	820(87.8%)	359(87.7%)	461(87.8%)	
Same	81(8.7%)	35(8.6%)	46(8.8%)	
Worse	33(3.5%)	15(3.7%)	18(3.4%)	0.98

* P-value generated using X^2^ test.

Info on some variables was missing or not applicable, some totals may be less than 939.

Nearly two thirds of participants (59.2%) were married or cohabiting, 66.3% had known their HIV status for over 2 years. Five percent of the PLHIV reported that they have never had children and of those who have ever had children, 7.5% had become childless. About 31.3% desired to have children and majority (80.3%) of these desired 1 or more children.

Table 2 shows the sexual behaviors, disclosure and ART status among the self reported sexually active study subjects. Eighty nine (89%) of the PLHIV reported that their regular sexual partners were aware of their HIV status and more than a quarter (29.2%) needed assistance to disclose their status to their regular sexual partner(s). Meanwhile, majority (80.7%) of the PLHIV reported that they knew the HIV status of their sexual partners. Over two thirds (76.5%) of PLHIV said that their regular sexual partners were HIV positive, 18.4% said their partners were negative and 25% declined to respond. Over 41% reported using condoms consistently over the past 12 months and a significant proportion (22.4%) never used condoms in the past 12 months.

**Table 2:** Sexual behaviors, disclosure and ART status among the sexually active.

Characteristics	n	Male	Female	P value*
**Regular sexual partner aware of HIV status of PLHIV**				
Yes	623(89.1%)	329(93.5%)	294(84.7%)	
No/don't know	76(10.9%)	23(6.5%)	53(15.3%)	<0.0001
**Did you need assistance to disclose your HIV status to regular sexual partner?**				
Yes	143(29.2%)	74(29.3%)	69(29.1%)	
No	347(70.8%)	179(70.7%)	168(70.9%)	0.97
**Who disclosed your HIV status to your regular sexual partner?**				
Self	352 (67.4%)	184 (63.9%)	168 (71.9%)	
Health care provider	152 (29.1%)	95 (33.0%)	57 (24.4%)	
Other	18 (3.5%)	9 (3.1%)	9 (3.8%)	0.09
**PLHIV aware of HIV status of their regular sexual partner(s)**				
Yes	566(80.7%)	317(90.3%)	249(71.1%)	
No/don't know	135(19.3%)	34(9.7%)	101(28.9%)	<0.0001
**HIV status of regular sexual partner(s)**				
Positive	375(76.5%)	221(78.7%)	154(73.7%)	
Negative	90(18.4%)	49 (17.4%)	41(19.6%)	
Declined response	25(5.1%)	11(3.9%)	14(6.7%)	0.28
**PLHIV ART status**				
Naïve	412(56.6%)	227(62.5%)	185(50.7%)	
Experienced	316(43.4%)	136(37.5%)	180(49.3%)	0.001
**Condoms use in past 12 months**				
Consistent	300 (41.3%)	165(45.2%)	135(37.3%)	
Inconsistent	264 (36.3%)	142(38.9%)	122(33.7%)	
Never	163 (22.4%)	58(15.9%)	105(29.0%)	<0.0001
**Having condoms at home**				
Yes	474 (65.1%)	258 (70.7%)	216 (59.5%)	
No	254 (34.9%)	107 (29.3%)	147 (40.5%)	0.002
**Have you been pregnant in the past 12 month (females?)**				
Yes	57(15.9%)	-	57(15.9%)	
No	302(84.1%)	-	302(84.1%)	-
**Was the pregnancy intended?**				
Yes	31(59.6%)	-	31(59.6%)	
No	21(40.4%)	-	21(40.4%)	-
**Have you caused a pregnancy in the past 12 months (Males)?**				
Yes	42(12.4%)	42(12.4%)	-	
No	297(87.6%)	297(87.6%)	-	-
**Was the pregnancy caused intended?**				
Yes	29(78.4%)	29(78.4%)	-	
No	8(21.6%)	8(21.6%)	-	-

* P-value generated using X^2^ test.

Info on some variables was missing or not applicable.

Among the females, 15.9% reported being pregnant within the past 12 months preceding the study. Of those who reported being pregnant, 59.6% said their pregnancies were desired. On the other hand, 12.4% of male subjects reported that they had caused a pregnancy within the past 12 months preceding the study. Of those who caused the pregnancy, 78.4% intended to cause the pregnancy.

### Sexual behaviors among PLHIV

The overall prevalence of safer sexual behavior (defined as reported abstinence or consistent condom use in the past 12 months was 54.1% (508/939)] and did not differ by gender (51.3% in males versus 56.3% in females, p = 0.131). Over 77% (731/939) of PLHIV were sexually active, 20% (146/731) of the sexually active PLHIV had 2+ sexual partners, 16.1% (108/671) had both regular and irregular sexual partners and 4.9% had only irregular sexual partners in the past 12 months preceding the study.

### Prevalence of and factors associated with consistent condom use

As shown in table 3, the prevalence of consistent condom use was 41.3% (300/731). It was significantly higher in males [45.2(165/365)] than in females [37.3 %( 135/362) P = <0.027].

**Table 3:** Prevalence, unadjusted and adjusted prevalence ratios (PRs) of factors associated with consistent condom use in past 12 months among HIV positive clients attending CSO health facilities in Uganda.

Characteristic	Consistent condom use % (n/N)	Unadjusted PRs (95% CI)	P value	Adjusted PRs (95% CI)	P value
**All**	41.3% (300/731)				
**Age** (years)					
18–24	32.8% (19/58)	1 (ref)		1 (ref)	
25–29	31.7% (32/101)	0.95 (0.59, 1.51)	0.824	0.70 (0.41, 1.18)	0.181
30–39	37.2% (106/285)	1.13 (0.76, 1.68)	0.555	0.71 (0.44, 1.15)	0.171
40–49	51.2% (108/211)	1.56 (1.05, 2.31)	0.025	0.78 (0.47, 1.30)	0.347
50+	48.6% (35/72)	1.49(0.96, 2.30)	0.078	0.76 (0.44, 1.33)	0.351
**Sex**					
Female	37.3% (135/362)	1 (ref)		1 (ref)	
Male	45.2% (165/365)	1.22 (1.02, 1.45)	0.027	0.99 (0.79, 1.23)	0.945
**Highest education level attained**					
None	50.9% (27/53)	1 (ref)		1 (ref)	
Primary	40.1% (176/439)	0.97 (0.77, 1.19)	0.696	0.68 (0.51, 0.92)	0.012
Secondary	38.3% (69/180)	1.28 (0.96, 1.70)	0.090	0.64 (0.45, 0.89)	0.010
University/tertiary	50.9% (27/53)	1.28 (0.96, 1.71)	0.094	0.86 (0.58, 1.27)	0.467
**Religion**					
Protestant/Anglican	33.6% (122/280)	1 (ref)			
Roman Catholic	40.7% (131/322)	1.06 (0.87, 1.27)	0.571	-	
Islam	41.2% (21/51)	1.01 (0.71, 1.44)	0.947	-	
Other	35.1% (26/74)	0.86 (0.61, 1.21)	0.393	-	
**Regular sexual partners aware of HIV status of PLHIV**					
No	22.4 %( 17/76)	1(ref)		1(ref)	
Yes	44.0 %( 274/623)	1.97(1.28–3.01)	0.002	0.63(0.34, 1.18)	0.138
**PLHIV aware of HIV status of regular sexual partner**					
No	25.2% (34/135)	1(ref)		1(ref)	
Yes	45.2(256/566)	1.80(1.32–2.44)	<0.0001	1.21(0.44, 3.32)	0.707
**HIV status of regular sexual partner**					
Positive	44.5 %( 167/375)	1(ref)		1(ref)	
Negative	58.9 %( 53/90)	1.32(1.07–1.63)	0.008	1.29(1.05, 1.57)	0.012
**Desire for more children**					
Yes	34.2% (88/257)	1 (ref)		1 (Ref)	
No	45.4% (210/463)	1.31 (1.08, 1.60)	0.007	1.17(0.93, 1.47)	0.171
**Marital status**					
Married/Living together	41.7% (225/540)	1 (ref)			
Divorced/Separated	37.8% (37/98)	0.89 (0.67, 1.17)	0.404	-	
Widowed/Widower	48.2% (27/56)	1.14 (0.85, 1.52)	0.382	-	
Never Married	33.3% (11/33)	0.8 0(0.49, 1.31)	0.379	-	
**Consumed alcohol in past 12 months**					
Yes	31.9% (76/238)	1 (ref)		1 (ref)	
No	45.8% (224/489)	1.42 (1.15, 1.75)	0.001	1.30 (1.03, 1.63)	0.024
**Duration since knowing one**'**s HIV results**					
<1year	23.2% (29/125)	1 (ref)		1 (ref)	
1–2 years	41.7% (68/163)	1.80 (1.25, 2.60)	0.002	1.17 (0.76, 1.78)	0.466
2.1–5 years	40.6% (78/192)	1.76 (1.23, 2.54)	0.002	1.14 (0.75, 1.71)	0.540
> 5 years	50.6% (125/247)	2.18 (1.55, 3.07)	<0.001	1.20 (0.80, 1.79)	0.393
**On ARVs**					
No	32.6% (103 /316)	1 (ref)		1(ref)	
Yes	47.3% (195/412)	1.45 (1.202, 1.75)	<0.0001	1.14 (0.92, 1.44)	0.202
**Attendance of HIV Prevention Discussion /support group**					
Yes	47.4% (189 /400)	1 (ref)		1(ref)	
No	33.8% (111/331)	0.70 (0.59, 0.85)	<0.001	0.90 (0.68, 1.18)	0.467
**Education and/or counselling on protecting self from another HIV strain**					
No	18.7% (17/91)	1 (ref)		1(ref)	
Yes	44.2% (283/640)	2.36 (1.52, 3.66)	<0.001	1.84 (1.08, 3.12)	0.025
**Skills**' **building workshops on safer sex behaviours such as (condom use, negotiation skills)**					
Yes	49.7% (184 /370)	1 (ref)		1(ref)	
No	32.5% (116/357)	0.65(0.54, 0.78)	<0.0001	0.80 (0.64, 1.01)	0.060

At bivariate analysis consistent condom use was higher among: people aged 40–49 years [PR 1.56, CI 1.05–2.31, P = 0.025]; males [PR 1.22, CI 1.02–1.45, P = 0.027]; PLHIV who reported that their regular sexual partners were aware of their HIV status [PR 1.97, CI 1.28–3.01, P = 0.002]; PLHIV who reported that they were aware of the HIV status of their regular sexual partner [UPR 1.80, CI 1.32–2.44, P = <0.0001]; PLHIV whose regular sexual partner's HIV status was negative [PR 1.32, 1.07–1.63, P = 0.008];PLHIV who never desired more children [PR 1.31, CI 1.08,−1.60), p = 0.007]; those who did not consume alcohol [PR 1.42, CI 1.15–1.75, P = <0.001]; those who had known their HIV status for more than one year; and those who were ART experienced [PR 1.45, CI 1.20–1.75, P = <0.001]. Those who had not attended HIV prevention discussion group [PR 0.70, CI0.59–0.85P = <0.001] and those who had not received counselling on prevention of acquisition of another strain of HIV [PR 0.42, CI 0.27–0.65, P = <0.001] had reduced prevalence ratios of using condoms consistently.

The independent predictors of higher likelihood of consistent use of condoms after adjusting for significant variables were HIV status of regular sexual partner being negative [aPR 1.29, CI 1.05–1.59, P = 0.012], not using alcohol [aPR  = 1.38, 1.12–1.70, P = 0.002], and having been counselled about protection of self from another strain of HIV [aPR 1.84, CI 1.08–3.12, P = 0.025].

### Prevalence of and factors associated with abstinence

As shown in table 4, the prevalence of abstinence was 22.1% (208/939).The proportion of abstinence increased with age from 9.4% in 18–24 year-olds to 40.5% among those aged 50 years or more. At unadjusted prevalence, abstinence was significantly higher among; females [30.6 %( 161/526). P = <0.0001]; those with no education [43.0 %( 40/93). P = 0.002]; those who desired not to have more children [27.0% (173/640). P = <0.0001]; those who did not take alcohol in past 12 months [26.2% (175/668). P = <0.0001]; those who knew their HIV status for more than 5 years [27.9% (104/373). P = 0.001]; and those who never attended discussion/support groups for HIV prevention [25.6% (112/445). P = 0.016].

**Table 4:** Prevalence, unadjusted and adjusted prevalence ratios (PRs) of factors associated with abstinence in past 12 months among HIV positive clients attending CSO health facilities in Uganda.

Characteristics	Abstinence % (n/N)	Unadjusted PRs (95% CI)	P value	Adjusted PRs (95% CI)	P value
**All**	22.1% (208/939)				
**Age** (years)					
18–24	9.4% (6/64)	1 (ref)		1 (ref)	
25–29	14.2% (17/120)	1.51 (0.63, 3.64)	0.36	2.0 (0.83, 4.88)	0.12
30–39	18.9% (67/354)	2.02 (0.91, 4.45)	0.08	3.01 (1.33, 6.79)	0.008
40–49	24.6% (69/280)	2.68 (1.19, 5.78)	0.016	3.23 (1.42, 7.35)	0.005
50+	40.5% (49/121)	4.32 (1.96, 9.53)	<0.0001	4.94 (2.17, 11.2)	<0.0001
**Sex**					
Male	11.4% (47/413)	1 (ref)		1 (ref)	
Female	30.6% (161/526)	2.69 (1.99, 3.62)	<0.0001	1.38 (1.03, 1.85)	0.028
**Highest education level**					
University/tertiary	12.7% (8/63)	1 (ref)		1 (ref)	
Secondary	15.0% (32/213)	1.18 (0.54, 2.57)	0.671	1.16 (0.62, 2.18)	0.623
Primary	22.5% (128/570)	1.77 (0.86, 3.61)	0.118	1.63 (0.90, 2.93)	0.102
None	43.0% (40/93)	3.39 (1.58, 7.23)	0.002	2.19 (1.20, 4.01)	0.010
**Religion**					
Protestant/Anglican	19.5% (69/353)	1 (ref)			
Roman Catholic	25.8% (112/434)	1.32 (1.01, 1.72)	0.04	-	
Islam	21.5% (14/65)	1.10 (0.66, 1.83)	0.71	-	
Other	14.9% (13/87)	0.76 (0.44, 1.32)	0.33	-	
**Desire for more children**					
Yes	11.7% (34/291)	1 (ref)		1 (ref)	
No	27.0% (173/640)	2.31 (1.65, 3.25)	<0.0001	1.35 (0.93, 1.96)	0.111
**Marital status**					
Married/Living together	2.5% (14/555)	1 (ref)		1 (ref)	
Divorced/Separated	40.1% (67/167)	15.9 (9.18, 27.5)	<0.0001	16.6 (9.34, 29.4)	<0.0001
Widowed/Widower	65.4% (108/165)	25.9 (15.3, 44.0)	<0.0001	17.8 (10.0, 31.8)	<0.0001
Never Married	35.3% (18/51)	14.0 (7.40, 26.4)	<0.0001	21.7 (11.5, 41.0)	<0.0001
**Alcohol in past 12 months**					
Yes	12.2% (33/271)	1 (ref)		1 (ref)	
No	26.2% (175/668)	2.15 (1.52, 3.03)	<0.0001	1.26 (0.90, 1.77)	0.173
**Duration since knowing one's HIV results**					
<1year	11.9% (17/143)	1 (ref)	1 (ref)		
1–2 years	19.9% (35/176)	1.67 (0.94, 2.98)	0.082	1.34 (0.85, 2.12)	0.204
2.1–5 years	21.1% (51/242)	1.77 (1.02, 3.07)	0.041	1.37 (0.89, 2.10)	0.147
>5 years	27.9% (104/373)	2.34 (1.40, 3.92)	0.001	1.39 (0.91, 2.14)	0.124
**On ARVs**					
No	17.6% (68 /387)	1 (ref)		1 (ref)	
Yes	25.4% (140/552)	1.44 (1.11, 1.87)	0.005	1.28 (1.01, 1.63)	0.036
**Attendance of HIV Prevention discussion/support group**					
Yes	19.0% (94 /494)	1 (ref)		1 (ref)	
No	25.6% (112/445)	1.35 (1.06, 1.71)	0.016	1.07 (0.81, 1.43)	0.607

After adjustment of variables that were significant on bivariate analysis, abstinence was more likely if a PLHIV was between 40 and 49 years [aPR 3.23,CI 1.42–7.35 p = 0.005]; 50 years and above [aPR 4.94,CI 2.17–11.2 p<0.0001]; had no education [aPR 2.19,CI 1.20–4.01 p = 0.010]; was never married [aPR 21.7, CI 11.5–41.0 p<0.001]; was divorced or separated [aPR 16.6, CI 9.34–29.4 p<0.001]; was widowed [aPR 17.8, CI 10.0–31.8, p<0.001]; and ART experienced [aPR 1.28, CI 1.01–1.63, p = 0.036]. Attendance of HIV discussion group, religion, sex and desire for children did not predict abstinence after multivariable adjustment.

## Discussions

The study highlights the prevalence and predictors of safer sexual behaviors among PLHIV receiving health care in CSOs supported by the CSF. More than 77% of the people living with HIV were sexually active in the past 12 months preceding the study. This is a high proportion compared to previous studies conducted in Uganda [12], [23] and elsewhere within the East African region [15], [16]. The increase in sexual activity among PLHIV could be attributed to improved quality of life because of antiretroviral therapy, opportunistic infection prophylaxis and other health care packages. With increased access to ART made possible by global and national initiatives, more HIV positive people are increasingly becoming healthier and more sexually active [24].

Improvement in sexual health for HIV positive people creates a dilemma between observance of sexual rights for these people and public health responsibility to prevent and control HIV transmission in communities. It was however encouraging noting that a fifth of the Study participants had abstained from sex, although these were more likely to be those study subjects who were 40 years and above indicative of decreasing sexual activity with age. Of those who were sexually active, majority (77.6%) used condoms and that 41.3% of them used them consistently. These findings are however lower compared to those in an urban clinic in Uganda [12]. More so 22.4% of those who were sexually active never used condoms at all and of those who used condoms, over 36% inconsistently used them. While we interpret these results with caution, the results appear to indicate overall reduction in consistent condom use among the PLHIV compared to a study by Tumukunde et al in Uganda [12]. The findings strengthen the available evidence that suggests that PLHIV are increasingly practicing risky sexual behaviors in Uganda and elsewhere in Sub-Saharan Africa [2], [25].

Further analysis of sexual practices revealed that 15.9% of females had become pregnant and 12.3% of males had caused pregnancies respectively. Similar findings have been reported before [12], [26].This data shows that almost 60% of the females reported that their pregnancies were desired and over 78% males who caused the pregnancy reported that they intended to cause the pregnancy.

These findings are of public health concern given that the study population was of HIV positive individuals likely to transmit new infection to their sexual partners, the unborn children or even re-infection with new HIV strains [8]. Over 15% of PLHIV had two or more sexual partners and over 18% of regular sexual partners were HIV negative. A combination of these factors could be a plausible explanation for the resurging HIV prevalence [11], [27].

When ART experienced study subjects were compared with ART naïve study participants, the ART experienced were more likely than the naïve ones to have engaged in safer sexual behavior. Irrespective of ART status, risky sexual behavior is of concern given that PLHIV form a reservoir for HIV infection.

In the present study, factors that predicted consistent condom use after controlling for confounding were: alcohol consumption, HIV status of regular sexual partner, and having received counseling on protecting self from other HIV strains.

These findings have significant implication on policy and practice. When PLHIV have received counseling on risk reduction through counseling, they are expected to be aware of the negative consequences of unprotected sex [28]. However, some PLHIV desire to have children [12] and the desire for children appears to be a driving force for risky sexual behavior [12], [28]. It is not surprising that risky sexual behavior was strongly associated with the married. In Uganda and most parts of Sub-Saharan Africa, social and cultural contexts put pressure on couples to produce children [25], [29], a demand that married couples have to fulfill. Being HIV positive and childlessness causes double stigma [25]. Childlessness causes even stronger stigma especially among married couples in most of sub-Saharan Africa [25]. When PLHIV are living quality lives, they oblige to societal demands to produce children. As a result, they succumb to unprotected sex but also overcome the stigma due to childlessness. In Togo, a study reported that many Study participants refused to use condoms because they desperately wanted to have children [29]. The authors illustrated that the desire of having children and overcoming social stigma, took precedence over the risk of infecting others or being re-infected [29].

Discordant couples in particular pose a special dilemma [28], [30]. One fact is that ART reduces risk of HIV transmission [30]. Building on this fact, it would be more beneficial to prioritize them for ART particularly those who desire children as this would minimize the risk of mother to child transmission and transmission of resistant strains. Higher uptake of PMTCT has a dual benefit of producing healthier babies and reducing the reservoir population [31].

As observed elsewhere [32], alcohol consumption was significantly associated with increased risky sexual practices. Those who reported alcohol consumption had reduced prevalence ratios of using condoms consistently and abstaining in the past 12 months. There is need for designing comprehensive but accessible programs that involve educating communities and HIV positive people in particular about alcohol abuse and its consequences.

This study was aimed at estimating the prevalence and factors associated with safer sexual behaviors. The strengths of the research design were a wider geographical scope that covered many parts of the country, and the big sample size. Nevertheless, this study had some limitations. Study participants were required to recall some issues that happened in the past. Thus, it is possible that recall bias compromised the accuracy of information that we collected. This bias could have been minimal given abstinence or consistent use of condoms is less likely to be forgotten with in a spell of one year. The topic of safer sexual behavior is a sensitive issue especially in the context of HIV. It is therefore worth noting that social desirability may have had some influences on our findings leading to overestimation of abstinence as well as condom use and underestimating desire for children as well as pregnancy. In addition, translating the study tool into multiple languages could have impacted on the validity of the tool despite the fact that we back translated to minimize the bias. Given that this was a cross sectional study, causal inferences could not be drawn. Nevertheless, this study provides useful information which is of practical importance to inform prevention programs using HIV positives in care.

## Conclusions

About half of PLHIV in care in Uganda are practicing safer sexual behaviors. Abstinence is more likely in those aged 40 years and above and is less likely among married and cohabiting people. Consistent condom use is predicted by alcohol drinking, HIV status of regular sexual partner and having attended a counseling session to prevent acquisition of new HIV strains. More than one third of the PLHIV desire more children and two thirds of pregnancies among PLHIV are intended.

In light of the high prevalence of sexual activity and risky sexual practices among PLHIV in care amidst increasing HIV incidence, these findings underscore the need for more efforts to improve positive prevention programming in Uganda. Uganda chronic HIV care and positive prevention guidelines and programs should lay emphasis on the need to counsel PLHIV in care about risk reduction during routine care and these should be tailored to address specific needs of PLHIV. Specifically, issues that should be addressed or given more attention in terms of resources, training and job aides should include: counseling guidelines should be designed to embrace child desire demands of PLHIV and those PLHIV who desire more children be prioritized for ART; counseling should emphasize messages about the need to protect oneself from re-infection with new HIV strains; alcohol abuse screening and treatment should be integral of the routine HIV care package and attendance in an HIV Prevention support group should be part of the risk reduction counseling methods.

## Supporting Information

Figure S1 Map showing zonal distribution of CSF sub-grantees in Uganda as of July 2010.
(TIF)

FigureS2 Illustrates sampling of study subjects at a CSO facility.
(TIF)

## References

[1] WHO (2011) Global HIV/AIDS response: epidemic update and health sector progress towards universal access: progress report 2011.

[2] Asiimwe-Okiror G, Opio AA, Musinguzi J, Madraa E, Tembo G, et al (1997) Change in sexual behaviour and decline in HIV infection among young pregnant women in urban Uganda. Aids 11: 1757–1763. 9386811 10.1097/00002030-199714000-00013

[3] Kirungi WL, Musinguzi J, Madraa E, Mulumba N, Callejja T, et al (2006) Trends in antenatal HIV prevalence in urban Uganda associated with uptake of preventive sexual behaviour. Sex Transm Infect 82: i36–41. 16581758 10.1136/sti.2005.017111 PMC2593075

[4] Berry S, Noble R (2006) Why is Uganda Interesting? Avert.org. Retrieved on 12 November 2013, from http://www.heart-intl.net/HEART/050107/WhyisUganda.htm.

[5] UAC (2011) Uganda AIDS Indicator Survey 2011 Preliminary Report March 2012. Kampala Uganda.

[6] Shafer LA, Biraro S, Nakiyingi-Miiro J, Kamali A, Ssematimba D, et al (2008) HIV prevalence and incidence are no longer falling in southwest Uganda: evidence from a rural population cohort 1989–2005. Aids 22: 1641–1649 doi: 1610.1097/QAD.1640b1013e32830a37502 18670225 10.1097/QAD.0b013e32830a7502

[7] UNAIDS (2012) Global AIDS Response Progress Report: Uganda Jan 2010-Dec 2012 Geneva.

[8] Signorelli D, Joseph RJ 2nd (1998) Epidemiology of HIV and AIDS. A retrospective look. Clin Podiatr Med Surg 15: 179–187. 9576048

[9] Allen C, Mbonye M, Seeley J, Birungi J, Wolff B, et al (2011) ABC for people with HIV: responses to sexual behaviour recommendations among people receiving antiretroviral therapy in Jinja, Uganda. Cult Health Sex 13: 529–543. 21390948 10.1080/13691058.2011.558593 PMC3082777

[10] Kennedy CE, Medley AM, Sweat MD, O'Reilly KR (2010) Behavioural interventions for HIV positive prevention in developing countries: a systematic review and meta-analysis. Bull World Health Organ 88: 615–623 doi: 610.2471/BLT.2409.068213. Epub 062010 May 068228 20680127 10.2471/BLT.09.068213 PMC2908966

[11] UAC (2010) National HIV Prevention Strategy 2011–15 for Uganda. Kampala Uganda. Kampala: Uganda AIDS Commission.

[12] Tumukunde D, Nuwaha F, Ekirapa E, Kityo C, Ssali F, et al (2010) Sexual behaviour among persons living with HIV/AIDS in Kampala, Uganda. East Afr Med J 87: 91–99. 23057304 10.4314/eamj.v87i3.62194

[13] Wandera B, Kamya MR, Castelnuovo B, Kiragga A, Kambugu A, et al (2011) Sexual behaviors over a 3-year period among individuals with advanced HIV/AIDS receiving antiretroviral therapy in an urban HIV clinic in Kampala, Uganda. J Acquir Immune Defic Syndr 57: 62–68 doi: 10.1097/QAI.1090b1013e318211b318213f318212 21297481 10.1097/QAI.0b013e318211b3f2 PMC3125399

[14] Chakrapani V, Newman PA, Shunmugam M, Dubrow R (2010) Prevalence and contexts of inconsistent condom use among heterosexual men and women living with HIV in India: implications for prevention. AIDS Patient Care STDS 24: 49–58 doi: 10.1089/apc.2009.0214 20095889 10.1089/apc.2009.0214 PMC2859766

[15] Sarna A, Luchters S, Pickett M, Chersich M, Okal J, et al (2012) Sexual behavior of HIV-positive adults not accessing HIV treatment in Mombasa, Kenya: Defining their prevention needs. AIDS Res Ther 9: 1742–6405. 10.1186/1742-6405-9-9 PMC3342087 22429560

[16] Sarna A, Luchters SMF, Geibel S, Kaai S, Munyao P, et al (2008) Sexual risk behaviour and HAART: a comparative study of HIV-infected persons on HAART and on preventive therapy in Kenya. International Journal of STD & AIDS 19: 85–89. 18334059 10.1258/ijsa.2007.007097

[17] Diabate S, Alary M, Koffi CK (2007) Determinants of adherence to highly active antiretroviral therapy among HIV-1-infected patients in Cote d'Ivoire. Aids 21: 1799–1803. 17690579 10.1097/QAD.0b013e3282a5667b

[18] R M, Jayanna K, Washington R, Das A, Pise G, et al (2011) Understanding positive prevention practices among people living with HIV in Karnataka, Southern India. Australas Med J 4: 150–161 doi: 110.4066/AMJ.2011.4577 Epub 2011 Apr 4030 23393506 10.4066/AMJ.2011.577 PMC3562893

[19] MPFED (2012) Key facts on Uganda's Population. Population Secretariate, Ministry of Finance, Planning and Economic Development. Kampala.

[20] MoH (2011) Status of Antiretroviral Therapy Services in Uganda. Kampala: Ministry of Health, Uganda.

[21] Kish L (1965) Survey sampling: John Willey and Sons, Inc. NY.

[22] Uganda 2006; Results from the demographic and health survey. Stud Fam Plann 40: 161–166. 10.1111/j.1728-4465.2009.00199.x 19662808

[23] Bateganya M, Colfax G, Shafer LA, Kityo C, Mugyenyi P, et al (2005) Antiretroviral therapy and sexual behavior: a comparative study between antiretroviral- naive and -experienced patients at an urban HIV/AIDS care and research center in Kampala, Uganda. AIDS Patient Care STDS 19: 760–768. 16283836 10.1089/apc.2005.19.760

[24] Matovu SN, La Cour K, Hemmingsson H (2012) Narratives of Ugandan Women Adhering to HIV/AIDS Medication. Occup Ther Int 19: 176–184 doi: 110.1002/oti.1330 Epub 2012 Jun 1027 22740312 10.1002/oti.1330

[25] Ncube NM, Akunna J, Babatunde F, Nyarko A, Yatich NJ, et al (2012) Sexual risk behaviour among HIV-positive persons in Kumasi, Ghana. Ghana Med J 46: 27–33. 22605886 PMC3353501

[26] Schackman BR, Dastur Z, Ni Q, Callahan MA, Berger J, et al (2008) Sexually active HIV-positive patients frequently report never using condoms in audio computer-assisted self-interviews conducted at routine clinical visits. AIDS Patient Care STDS 22: 123–129 doi: 110.1089/apc.2007.0037 18260803 10.1089/apc.2007.0037

[27] Amirkhanian YA, Kelly JA, Kabakchieva E, Kirsanova AV, Vassileva S, et al (2005) A randomized social network HIV prevention trial with young men who have sex with men in Russia and Bulgaria. Aids 19: 1897–1905. 16227798 10.1097/01.aids.0000189867.74806.fb

[28] Nattabi B, Li J, Thompson SC, Orach CG, Earnest J (2009) A systematic review of factors influencing fertility desires and intentions among people living with HIV/AIDS: implications for policy and service delivery. AIDS Behav 13: 949–968 doi: 910.1007/s10461-10009-19537-y Epub 12009 Mar 10428 19330443 10.1007/s10461-009-9537-y

[29] Moore AR, Oppong J (2007) Sexual risk behavior among people living with HIV/AIDS in Togo. Social Science & Medicine 64: 1057–1066. 17101202 10.1016/j.socscimed.2006.10.004

[30] Biraro S, Ruzagira E, Kamali A, Whitworth J, Grosskurth H, et al (2013) HIV-1 transmission within marriage in rural Uganda: a longitudinal study. PLoS One 8: e55060 doi: 55010.51371/journal.pone.0055060 Epub 0052013 Feb 0055064 23390512 10.1371/journal.pone.0055060 PMC3563659

[31] Shah M, Johns B, Abimiku A, Walker DG (2011) Cost-effectiveness of new WHO recommendations for prevention of mother-to-child transmission of HIV in a resource-limited setting. Aids 25: 1093–1102 doi: 1010.1097/QAD.1090b1013e32834670b32834679 21505317 10.1097/QAD.0b013e32834670b9

[32] Saniel OP, De los Reyes SJ (2010) Prevalence of risky behaviours and determinants of multiple sex partnerships among male Filipino seafarers. Int Marit Health 62: 215–223. 21348015

