# Supplementary figures and images for: Sexual Behavior among Persons Living with HIV in Uganda: Implications for Policy and Practice
Source: PLoS One. 2014 Jan 23;9(1):e85646. doi: 10.1371/journal.pone.0085646 (PMC3900429; doi:10.1371/journal.pone.0085646)

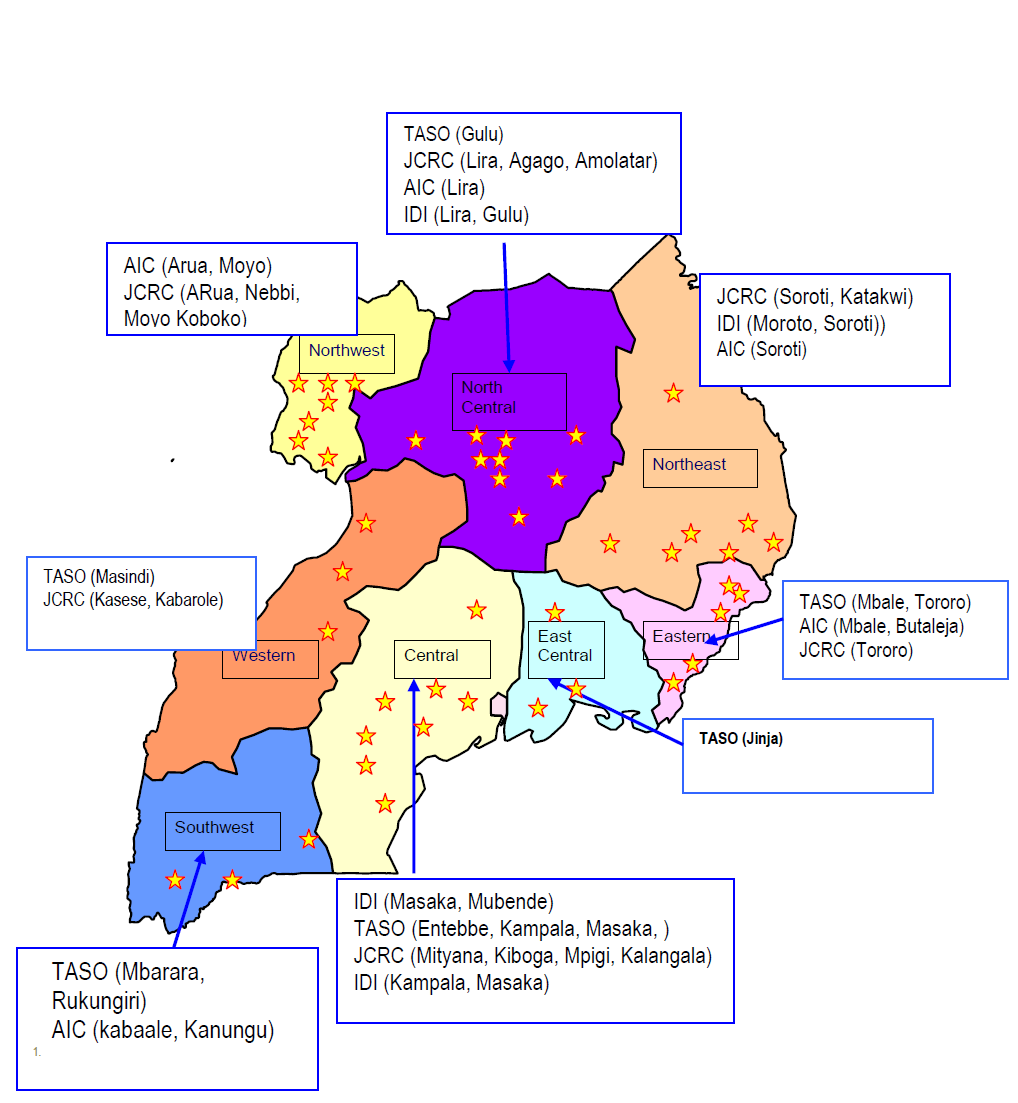

Supplement: Figure S1 — Map showing zonal distribution of CSF sub-grantees in Uganda as of July 2010. (TIF) [file pone.0085646.s001.tif]

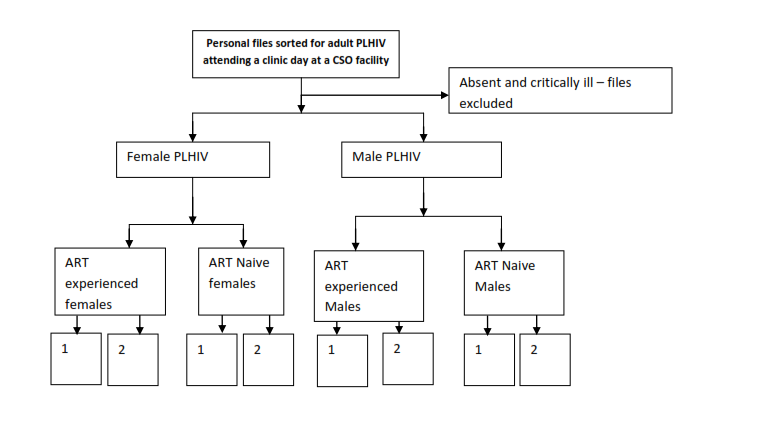

Supplement: FigureS2 — Illustrates sampling of study subjects at a CSO facility. (TIF) [file pone.0085646.s002.tif]
